# Clinical Factors Associated with Long-Term Survival in Metastatic Melanoma Treated with Anti-PD1 Alone or in Combination with Ipilimumab

**DOI:** 10.3390/curroncol29100608

**Published:** 2022-10-14

**Authors:** Siddhartha Goutam, Igor Stukalin, Benjamin Ewanchuk, Michael Sander, Philip Q. Ding, Daniel E. Meyers, Daniel Heng, Winson Y. Cheung, Tina Cheng

**Affiliations:** 1 Faculty of Medicine and Dentistry, University of Alberta, Edmonton, AB T6G 2R7, Canada; 2 Tom Baker Cancer Center, University of Calgary, Calgary, AB T2N 4N2, Canada; 3 Cumming School of Medicine, University of Calgary, Calgary, AB T2N 4N1, Canada; 4 Oncology Outcomes, Calgary, AB T2N 4N2, Canada

**Keywords:** immunotherapy, metastatic melanoma, immune checkpoint inhibitors, survival, long-term survival, short-term survival, pembrolizumab, nivolumab, ipilimumab

## Abstract

Immune checkpoint inhibitors (ICIs) for treatment of metastatic melanoma (MM) offer lasting overall survival (OS) benefit in a subset of patients. However, outcomes remain poor for non-responders. Clinical predictors of long-term survival remain elusive. We utilized the Alberta Immunotherapy Database to investigate the association of host and disease characteristics, and treatment factors with overall survival (OS) greater than 3 years. We identified patients treated between August 2013 and May 2020 with single-agent anti-PD1 or combination (anti-PD1 and anti-CTLA4) ICI regimens. A logistic regression model was used to assess for independent association between clinical factors captured and survival greater than 3 years. Statistically significant factors on univariable analysis were assessed using multivariable analysis. In total, 284 of 460 patients were identified to have short-term (<1 year) or long-term (>3 years) survival with 186 surviving <1 year and 98 surviving >3 years. The median age was 64 and 18.4% of patients were ECOG ≥ 2. On logistic regression, Breslow’s Depth ≤ 4 mm, normal serum LDH, normal serum albumin and M-stage 1a/b were associated with OS > 3 years on univariable and multivariable analysis. ECOG < 2, dNLR ≤ 3, normal hemoglobin were only associated with survival on the univariable analysis but not in the multivariable analysis. The objective response rate in long-term survivors was 83.7% compared to 7.5% in the short-term survivors. Our study identifies four easily accessible predictors of long-term survival in a large real-world MM cohort treated with ICI.

## 1. Introduction

Metastatic melanoma (MM) historically had a median survival of 6–9 months with 1-year survival rate at 25.5% [1]. In the past decade, two therapeutic strategies have significantly improved outcomes in MM: molecularly targeted therapy for BRAF-mutant melanoma and immune checkpoint inhibitors (ICIs) that are not mutation-specific. BRAF targeted therapy can induce rapid tumor response in BRAF-mutant melanoma and improve survival. Meanwhile, ICIs targeting cytotoxic T-lymphocyte associated protein 4 (CTLA-4), such as ipilimumab, and programmed death-1 (PD-1), such as nivolumab or pembrolizumab, alone or in combination can result in sustained tumor regression with the possibility of prolonged survival [2,3,4,5]. In BRAF-mutant MM, ICIs remain superior in delivering long-term survival benefit when compared to BRAF-targeted therapies and are most commonly chosen as first-line therapies [6]. Currently, first-line ipilimumab plus nivolumab demonstrated a remarkable 5 year survival rate at 52%, however 30% or patients progressed within six months, with much shorter survival [4]. 

Factors associated with long-term survival in melanoma treated with ICI remain not well understood. Recently, models to predict response and median overall survival for MM treated with ICI have been constructed using clinical parameters including Eastern Cooperative Oncology Group (ECOG) performance status, presence of liver and lung metastases, serum lactate dehydrogenase (LDH) level, blood neutrophil-lymphocyte ratio (NLR), type of therapy, and line of treatment [7]. One study found that the presence of liver or bone metastases was independently associated with reduced likelihood of survival at 5 years, whereas ECOG performance status of 0 was independently associated with an increased likelihood of 5-year survival in nivolumab-treated patients [8]. Of the 270 patients included in this study, only 107 (39.6%) had melanoma, with the remaining had renal cell carcinoma and non-small cell lung cancer. The purpose of our study is to identify factors associated with long-term survival of three or more years in MM patients treated with anti-PD1 agents alone or in combination with ipilimumab. 

## 2. Materials and Methods

### 2.1. Study Population and Design

Approval for this study was obtained through the Health Research Ethics Board of Alberta–Cancer Committee (ID 17-0125). We conducted a multicenter retrospective cohort study at two tertiary cancer centers (Tom Baker Cancer Centre, Calgary, Alberta; Cross Cancer Institute in Edmonton, Alberta) and four regional cancer centers (Central Alberta Cancer Center, Red Deer, Alberta; Grande Prairie Cancer Center, Grande Prairie, Alberta; Jack Ady Cancer Center, Lethbridge, Alberta, Margery E. Yiull Cancer Center, Medicine Hat, Alberta) in Canada.

Patients treated with anti-PD1 alone or in combination with ipilimumab in all lines of therapy were identified using consecutive provincial pharmacy records. Inclusion criteria for this study were: patients aged >18 years at the time of metastatic disease diagnosis, histologically confirmed melanoma, and initiation of ICI therapy (nivolumab, pembrolizumab, ipilimumab and nivolumab) between 1 January 2010 and 31 May 2020. Patients with ocular melanoma were excluded from analysis. The data collection and chart review process occurred between 1 July 2017 and 1 July 2021. The data were obtained through registry, pharmacy, and/or consecutive clinic lists by individual retrospective chart reviews using standardized database templates. We collected disease characteristics, clinical and biochemical parameters at baseline of treatment, date of first treatment, best radiographic response, length on treatment, and date of death or last follow-up. Clinical staging was based on the criteria of American Joint Committee on Cancer (AJCC) 8th edition [9]. Response assessments were performed by the treating physicians as per Response Evaluation Criteria in Solid Tumors (RECIST) version 1.1 [10] was used to determine treatment response. 

### 2.2. Outcomes of Interest

The primary outcome of interest was overall survival (OS) and time to treatment failure (TTF) in MM patients after receiving anti-PD1 alone or in combination with ipilimumab in any line of therapy, and clinical factors associated with long-term survival. OS was calculated from the date of treatment start until either the date of death from any cause or the date of last follow up for patients still alive at the time of data collection. The objective response rate (ORR) was defined as the proportion of patients achieving a complete or partial radiographic response during treatment with anti-PD1 alone or in combination with ipilimumab in any line of therapy based on RECIST v1.1 [10]. 

### 2.3. Statistical Analysis

Baseline demographic and clinical characteristics were analyzed using descriptive statistics. OS was plotted as a frequency histogram to visualize the distribution of survival time within the cohort. In order to assess factors associated with long-term survival, we divided patients into short-term versus long-term survivors. Survival <1 year was chosen as a cutoff for short-term survival, as this survivorship represents patients who derived minimal benefit from ICIs and their survival were not different from the historical prognosis [1]. Long-term survivor was defined as survival >3 years based on previous work demonstrating that patients who had survived for 3 years after starting on ICIs had greatly lowered risk of death after this point [4,5,11,12]. Patients whose survival were between 1 and 3 years were excluded for all subsequent analyses. The relationship between survival and patient factors between the two groups were evaluated using Pearson’s chi-squared test for categorical variables with all expected cell sizes ≥5, Fisher’s exact test for categorical variables with any expected cell size <5, and Wilcoxon rank sum test for continuous variables.

Univariate logistic regression was performed with key baseline characteristics to assess their independent associations with long-term survival. Characteristics with significant predictive value (*p* < 0.05) in the univariable analysis were then included in a multivariable logistic regression model to predict long-term survival. Missing data were handled by the case-deletion method. All analyses were conducted using R, a free software for statistical computing [13]. 

## 3. Results

### 3.1. Patient Characteristics

Among, 460 patients were identified with MM were treated with anti-PD 1 alone or in combination with ipilimumab. 303 (66%) of the patients were male and median age was 61. 144 (32%) patients harbored a BRAF mutation (V600E, V600K). Among 149 *BRAF*-mutant patients, 30 (20%) received an anti-PD-1 agent alone in the first-line, 23 (15%) received anti-PD-1 after ipilimumab, and 42 (28%) received an anti-PD-1 agent plus ipilimumab first-line. 76 (51%) patients received BRAF targeted therapies prior to ICIs, among them 37 (25%) received anti-PD1 alone as second line, 19 (13%) received an anti-PD-1 agent after prior ipilimumab, and 9 (6%) received an anti-PD-1 agent with ipilimumab in the second line. Among 311 patients without *BRAF* mutation, 154 (50%) received an anti-PD-1 agent alone in the first-line, 43 (14%) received anti-PD-1 alone after ipilimumab, and 45 (14%) received anti-PD-1 with ipilimumab first-line.

After exclusion of patients surviving 1–3 years, 284 (62%) patients remained. From this cohort, 98 (21%) patients survived >3 years and 186 (40%) patients survived <1 year.. Baseline clinical and pathological characteristics for this cohort are seen in Table 1. 

### 3.2. Survival and Efficacy

**Figure 1:**
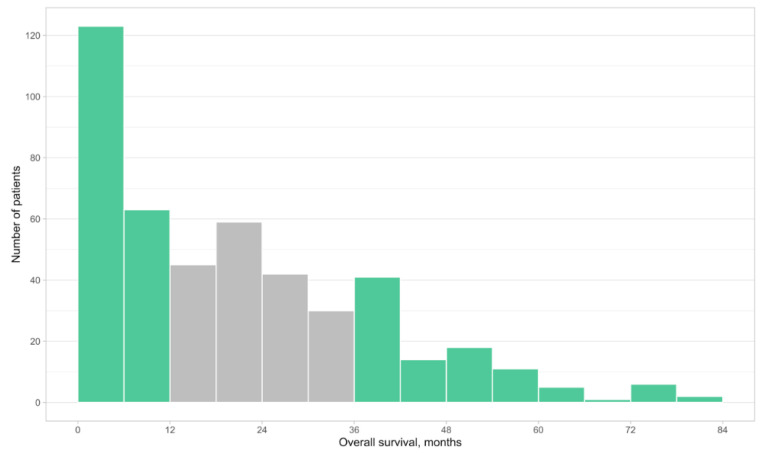
Histogram demonstrating pattern of survival in patients surviving <1 year and >3 years (in green). Patients who survived beyond 1 year but under 3 years are represented in grey (not included in the main study).

At the time of data cutoff, 30.6% of the patients were alive and the median follow-up was 43.6 months (95% CI: 3.68–81.35 months). The median OS of the entire cohort (*n = 460*) after initiation of ICIs was 18 months. Patient survival in months is seen as a frequency histogram in Figure 1. Among patients who survived under 1 year (*n = 186*), 123 (66%) survived under 6 months. Among patients who survived greater than 3 years, 63 (33.8%) survived beyond 5 years. The median time to treatment failure (TTF) of the entire cohort was 4 months. The objective response rate of the entire cohort was 56%.

In the patients that survived <1 year (*n =* 186), the median TTF was 1 month and the objective response rate was 7.5%. In patients surviving ≥3 years the median TTF was 13 months and the objective response rate was 83.7%. The proportion of complete response (CR), partial response (PR), stable disease (SD), and progressive disease (PD) in the OS < 1 year cohort were 4.0%, 10.1%, 12.1% and 73.7%. respectively. In the OS ≥ 3 years cohort they were 35.8%, 50.5%, 13.7%, and 0%, respectively (Table 2).

### 3.3. Logistic Regression

The univariable analysis (Table 3), consisted of 17 variables based on the demographic and clinical factors described. Age, sex, and BMI were not significantly associated with OS ≥ 3 years. ECOG PS ≥ 2 was associated with survival <1 year (OR = 0.28, 95% CI: 0.12–0.60). Breslow’s depth > 4 mm (OR:0.36, 95% CI: 0.18–0.70) and LDH > upper limit of normal (ULN) (OR:0.21, 95% CI: 0.10–0.39) were associated with OS < 1 year. Furthermore, derived neutrophil to lymphocyte ratio (dNLR) >3 (OR = 0.25, 95% CI: 0.11–0.49), hemoglobin < lower limit of normal (LLN) (OR = 0.27, 95% CI: 0.15–0.48), albumin < LLN (OR = 0.12, 95% CI: 0.04–0.26) and creatinine > ULN (OR = 0.44, 95% CI: 0.18–0.95) were associated with survival <1 year. Lastly, metastasis to liver, bone, and brain, presence of >3 metastatic sites and M1c/1d stage were associated with survival <1 year (Table 3).

In the multivariable analysis (Table 4) Breslow’s depth >4 mm (OR: 0.43, 95% CI: 0.18–0.97), LDH > ULN (OR: 0.37, 95% CI: 0.15–0.88), albumin < LLN (OR: 0.22, 95% CI: 0.06–0.67) and M1c/1d stage (OR: 0.30, 95% CI: 0.13–0.68) were independently associated with OS < 1 year. ECOG-PS ≥ 2, dNLR > 3, Hemoglobin < LLN and metastatic sites >3 were not independently associated with OS < 1 year.

## 4. Discussion

In this clinical series including both academic and community centers, we report survival outcomes and factors associated with long-term survival in 460 MM patients after receiving ICIs. At a median follow-up of 43.6 months, median OS of the entire cohort after initiation of ICIs was 18 months. Among them, 98 (21%) patients were long-term survivors (survived greater than 3 years) and 186 (40%) patients were short term survivors (survived less than 1 year). We identified the follow factors to be prognostic of long-term survival on the multivariable logistic regression: Breslow thickness ≤ 4 mm, normal LDH, normal Albumin, and M1a/1b stage. 

We chose survival greater than 3 years as the cut-off for long-term survival based on reported survival data. ICIs target the dysfunctional immune system to restore an adaptive host immune response against the malignancy, therefore delivering durable cancer control. Ipilimumab was the first ICI that demonstrated that durable melanoma response to treatment with long-term survival was possible. Over 20% of ipilimumab-treated MM may achieve long term survival, some for greater than 10 years [11,12]. Furthermore, response rate and quality of response improve over time without additional exposure to ipilimumab [12,14]. The above observations lead to the realization that ICI may have curative potential and the concept of a clinical cure in long-term melanoma survivals have been proposed [15]. Similar distinct flattening of the OS curves after 3 years, with a lower risk of death thereafter have been reported for anti-PD-1 with nivolumab or pembrolizumab alone or nivolumab in combination with ipilimumab. The pivotal phase III melanoma trial reported survival rate at 52% and 46% in nivolumab-treated and 58% and 52% in nivolumab plus ipilimumab-treated patients at 3 and 5-years, respectively [4,5]. As one major goal in treating MM is to increase the proportion of patients who can achieve durable survival through improved patient selection, it is therefore important to understand factors associated with long-term survival. It is also paramount to understand factors associated with short-term survival after receiving ICIs as this subset of patients derive minimum or no benefit from ICIs, for whom other therapeutic strategies are needed. 

M-stage 1a/1b was prognostic of favorable OS when compared to M1c/1d stages. This is consistent what is expected based on AJCC staging criteria. Previously, Byun et al. showed have showed that the absence of visceral organ metastasis is associated with greater overall survival [16]. The presence of non-lung visceral organ metastases elevates M-stage of the disease to M1c/1d [9]. Ku et al. showed that specifically metastasis to the liver were prognostic of worse outcomes while metastases to bone, lung and brain were not [17]. Our logistic regression did not analyze the association of metastatic sites with OS greater than 3 years. 

Baseline LDH has also been shown to be prognostic in many studies, and is now generally recognized as a prognostic factor [16,18,19,20,21,22,23]. This phenomenon held true in our study as well with elevated LDH found to be independently prognostic of OS under 1 year. Breslow’s depth was found to be prognostic, which has historically been used as a surrogate marker of disease extent in melanoma [9,22,24]. The biology underlying this finding is unknown and requires further study, however we postulate that greater Breslow’s depth is suggestive of more aggressive disease and/or a delay in diagnosis. An association between Breslow’s depth and survival has not been seen in the metastatic setting, in the context of ICI treatment. Lastly, normal albumin was found to be prognostic of long-term survival in this cohort on multivariable analysis. Low albumin has been described as associated with poor OS across multiple cancers however the studies of this factor in melanoma are sparse [25]. Albumin is a surrogate marker for a heightened inflammatory state; thus, a low albumin may correlate to a more advanced disease state or immune dysfunction. Ours is the first study to our knowledge to suggest an independent correlation between normal albumin and long-term survival in metastatic melanoma. 

Interestingly, ECOG performance status < 2 and DNLR ≤ 3 were found not found to be significant in the multivariable analysis. This is in contrast to the findings of Silva et al. who recently produced a multivariable prediction model for response to immune checkpoint inhibitors which included NLR as a continuous variable and ECOG performance status with a cutoff of ≥1 [7]. Unaccounted co-variability between ECOG performance status and other factors within our multivariable analysis may explain the lack of an independent association with survival in our analyses. dNLR is a marker of inflammation and has been described to be independently associated with worse survival when greater than or equal to 3. Capone et al. described the optimal cutoff to be at 3.8 [26]. Thus, it is possible that the cutoff of 3 in this population was not prognostic of short-term survival in this cohort. Additionally, anemia and elevated creatinine were not found to be associated with short term survival on multivariable analysis. Anemia has been previously described as associated with shorter survival but the biology underlying this finding is unknown [7,27].

Limitations of the study include its retrospective nature and thus unaccounted biases in patient and treatment selection as well as missing patient data. The study population included both first-line patients as well as those being treated at later lines of therapy. The use of subsequent treatments beyond progression that may affect survival was not taken into consideration. 

## 5. Conclusions

We identified four independent prognostic factors for long-term survival in MM patients treated with ICIs including Breslow’s depth ≤4 mm, normal LDH, normal albumin and M-stage 1a/1b. Collectively these factors may identify a population that are likely to see a significant survival benefit from treatment with ICI. Further research is needed to identify patients that will derive the most benefit from ICI treatment.

## Figures and Tables

**Table 1:** Baseline demographic and clinical characteristics of the patient cohort excluding patients with survival between 1–3 years (*n = 284*).

			Overall Survival (y)		
Characteristic	N	Cumulative, N = 284	<1, N = 186	>3, N = 98	*p* Value
**Age**	284	64 (54, 74)	65 (55, 76)	62 (52, 72)	0.10
<65		150 (52.8%)	91 (48.9%)	59 (60.2%)	0.07
≥65		134 (47.2%)	95 (51.1%)	39 (39.8%)	
**Sex**	284				0.19
Female		93 (32.7%)	56 (30.1%)	37 (37.8%)	
Male		191 (67.3%)	130 (69.9%)	61 (62.2%)	
**BMI** (kg/m^2^)	253	27 (24, 31)	27 (24, 32)	27 (24, 30)	0.49
<30		172 (68.0%)	104 (64.2%)	68 (74.7%)	0.09
≥30		81 (32.0%)	58 (35.8%)	23 (25.3%)	
**ECOG-PS**	283				**0.001**
<2		231 (81.6%)	141 (76.2%)	90 (91.8%)	
≥2		52 (18.4%)	44 (23.8%)	8 (8.2%)	
**Autoimmune condition**	283				0.36
No		244 (86.2%)	157 (84.9%)	87 (88.8%)	
Yes		39 (13.8%)	28 (15.1%)	(11.2%)	
**Melanoma Type**	284				0.20
Cutaneous		258 (90.8%)	166 (89.2%)	92 (93.9%)	
Mucosal		26 (9.2%)	20 (10.8%)	6 (6.61%)	
**BRAF Mutation**	253				0.12
No		161 (63.6%)	100 (60.2%)	61 (70.1%)	
Yes		92 (36.4%)	66 (39.8%)	26 (29.9%)	
**Breslow Thickness** (mm)	188	3.0 (1.8, 5.1)	3.6 (2.0, 6.0)	2.3 (1.5, 3.7)	**0.005**
≤4		117 (62.2%)	65 (54.2%)	52 (76.5%)	**0.002**
>4		71 (37.8%)	55 (45.8%)	16 (23.5%)	
**Ulceration**	178				0.17
No		78 (43.8%)	46 (40.0%)	32 (50.8%)	
Yes		100 (56.2%)	69 (60.0%)	31 (49.2%)	
**Mitotic Rate** (per mm^2^)	148				0.70
<1		7 (4.7%)	4 (4.2%)	3 (5.7%)	
≥1		141 (95.3%)	91 (95.8%)	50 (94.3%)	
**Lactate Dehydrogenase**	233				**<0.001**
Normal		147 (63.1%)	76 (51.4%)	71 (83.5%)	
High		86 (36.9%)	72 (48.6%)	14 (16.5%)	
**dNLR**	274				**<0.001**
≤3		207 (75.5%)	121 (68.0%)	86 (89.6%)	
>3		67 (24.5%)	57 (32.0%)	10 (10.4%)	
**Hemoglobin**	275				**<0.001**
Low		167 (60.7%)	91 (50.8%)	76 (79.2%)	
Normal		108 (39.3%)	88 (49.2%)	20 (20.8%)	
**Albumin**	241				**<0.001**
Low		173 (71.8%)	94 (60.3%)	79 (92.9%)	
Normal		68 (28.2%)	62 (39.7%)	6 (7.1%)	
**Creatinine**	265				**0.04**
Low		225 (84.9%)	143 (81.7%)	82 (91.1%)	
High		40 (15.1%)	32 (18.3%)	8 (8.9%)	
**Calcium**	240				0.39
Low/Normal		226 (94.2%)	143 (92.9%)	83 (96.5%)	
High		14 (5.8%)	11 (7.1%)	3 (3.5%)	
**M Stage**	284				**<0.001**
1a/1b		231 (81.3%)	140 (75.3%)	91 (92.9%)	
1c/1d		53 (18.7%)	46 (24.7%)	7 (7.1%)	
**Metastasis Sites**	284				**<0.001**
≤3 sites		129 (45.4%)	69 (37.1%)	60 (61.2%)	
>3 sites		155 (54.6%)	117 (62.9%)	38 (38.8%)	
**Lung Metastasis**	284				0.97
No		125 (44.0%)	82 (44.1%)	43 (43.9%)	
Yes		159 (56.0%)	104 (55.9%)	55 (56.1%)	
**Liver Metastasis**	284				**0.007**
No		191 (67.3%)	115 (61.8%)	76 (77.6%)	
Yes		93 (32.7%)	71 (38.2%)	22 (22.4%)	
**Bone Metastasis**	284				**0.02**
No		224 (78.9%)	139 (74.7%)	85 (86.7%)	
Yes		60 (21.1%)	47 (25.3%)	13 (13.3%)	
**Brain Metastasis**	284				**0.002**
No		223 (78.5%)	136 (73.1%)	87 (88.8%)	
Yes		61 (21.5%)	50 (26.9%)	11 (11.2%)	

**Table 2:** Objective response rate (ORR) as determined by Response Evaluation Criteria in Solid Tumors (RECIST).

Characteristic	N	All Patients	Overall Survival (y)		*p*-Value
			<1, N = 186	>3, N = 98	
**Objective Response Rate**	284	96 (33.8%)	14 (7.5%)	82 (83.7%)	**<0.001**
Best Response	194				**<0.001**
Complete Response (CR)		38 (19.6%)	4 (4.0%)	34 (35.8%)	
Partial Response (PR)		58 (29.9%)	10 (10.1%)	48 (50.5%)	
Stable Disease (SD)		25 (12.9%)	12 (12.1%)	13 (13.7%)	
Progressive Disease (PD)		73 (37.6%)	73 (73.7%)	0 (0.0%)	

Percentages reported based on total number of best responses known in each survival group.

**Table 3:** Univariable regression analysis of patient factors to determine association with survival >3 years or <1 year.

Characteristic	Odds Ratio	95% Confidence Interval	*p-*Value
**Age**			
<65 (reference) vs. ≥65	0.63	0.38, 1.04	0.07
**Sex**			
Female (reference) vs. Male	0.71	0.42, 1.19	0.19
**Body mass index, kg/m^2^**			
<30 (reference) vs. ≥30	0.61	0.34, 1.06	0.08
**ECOG performance status**			
<2 (reference) vs. ≥2	0.28	0.12, 0.60	**<0.001**
**Autoimmune condition**			
No (reference) vs. Yes	0.71	0.32, 1.46	0.36
**Melanoma type**			
Cutaneous (reference) vs. mucosal	0.54	0.19, 1.32	0.19
**BRAF mutation**			
No (reference) vs. Yes	0.65	0.37, 1.12	0.12
**Breslow thickness, mm**			
≤4 (reference) vs. >4	0.36	0.18, 0.70	**0.002**
**Ulceration**			
No (reference) vs. Yes	0.65	0.35, 1.20	0.17
**Mitotic rate, per mm^2^**			
<1 (reference) vs. ≥1	0.73	0.16, 3.84	0.69
**Lactate dehydrogenase**			
Normal (reference) vs. High	0.21	0.10, 0.39	**<0.001**
**Derived neutrophil to lymphocyte ratio**			
≤3 (reference) vs. >3	0.25	0.11, 0.49	**<0.001**
**Hemoglobin**			
Normal (reference) vs. Low	0.27	0.15, 0.48	**<0.001**
**Albumin**			
Normal (reference) vs. Low	0.12	0.04, 0.26	**<0.001**
**Creatinine**			
Normal (reference) vs. High	0.44	0.18, 0.95	**0.04**
**Calcium**			
Low/Normal (reference) vs. High	0.47	0.10, 1.56	0.23
**M stage**			
1a/1b (reference) vs. 1c/1d	0.37	0.22, 0.62	**<0.001**
**# of organ sites with metastasis**			
≤3 (reference) vs. >3	0.23	0.09, 0.51	**<0.001**
**Site of metastasis**			
Lung	1.01	0.62, 1.65	0.97
Liver	0.47	0.26, 0.81	**0.006**
Bone	0.45	0.22, 0.86	**0.02**
Brain	0.34	0.16, 0.67	**0.001**

**Table 4:** Multivariable analysis of patient factors associated with survival >3 years.

	Odds Ratio	95% Confidence Interval	*p-*Value
**ECOG performance status**			
<2 (reference) vs. ≥2	1.55	0.40, 6.01	0.52
**Breslow thickness, mm**			
≤4 (reference) vs. >4	0.43	0.18, 0.97	**0.04**
**Lactate dehydrogenase**			
Normal (reference) vs. High	0.37	0.15, 0.88	**0.02**
**Derived neutrophil to lymphocyte ratio**			
≤3 (reference) vs. >3	0.82	0.26, 2.45	0.73
**Hemoglobin**			
≥LLN (reference) vs. <LLN	0.62	0.23, 1.66	0.34
**Albumin**			
≥LLN (reference) vs. <LLN	0.22	0.06, 0.67	**0.007**
**Creatinine**			
Normal (reference) vs. High	1.00	0.28, 3.30	0.99
**M stage**			
1a/1b (reference) vs. 1c/1d	0.30	0.13, 0.68	**0.004**
**Number of organ sites with** **metastasis**			
≤3 (reference) vs. >3	0.71	0.22, 2.19	0.56

## Data Availability

Data will not be shared, as the ethics approval for this project does not allow for sharing of this data due to patient privacy and confidentiality.
